# Diagnosis of Alzheimer's Disease Based on Structural MRI Images Using a Regularized Extreme Learning Machine and PCA Features

**DOI:** 10.1155/2017/5485080

**Published:** 2017-06-18

**Authors:** Ramesh Kumar Lama, Jeonghwan Gwak, Jeong-Seon Park, Sang-Woong Lee

**Affiliations:** ^1^National Research Center for Dementia, Gwangju, Republic of Korea; ^2^Department of Software, Gachon University, 1342 Seongnamdaero, Sujeonggu, Seongnam, Gyeonggido 13120, Republic of Korea; ^3^School of Electrical Engineering and Computer Science (EECS), Gwangju Institute of Science and Technology (GIST), Gwangju 61005, Republic of Korea; ^4^Department of Multimedia, Chonnam National University, 50 Daehakro, Yeosu, Jeollanamdo 59626, Republic of Korea

## Abstract

Alzheimer's disease (AD) is a progressive, neurodegenerative brain disorder that attacks neurotransmitters, brain cells, and nerves, affecting brain functions, memory, and behaviors and then finally causing dementia on elderly people. Despite its significance, there is currently no cure for it. However, there are medicines available on prescription that can help delay the progress of the condition. Thus, early diagnosis of AD is essential for patient care and relevant researches. Major challenges in proper diagnosis of AD using existing classification schemes are the availability of a smaller number of training samples and the larger number of possible feature representations. In this paper, we present and compare AD diagnosis approaches using structural magnetic resonance (sMR) images to discriminate AD, mild cognitive impairment (MCI), and healthy control (HC) subjects using a support vector machine (SVM), an import vector machine (IVM), and a regularized extreme learning machine (RELM). The greedy score-based feature selection technique is employed to select important feature vectors. In addition, a kernel-based discriminative approach is adopted to deal with complex data distributions. We compare the performance of these classifiers for volumetric sMR image data from Alzheimer's disease neuroimaging initiative (ADNI) datasets. Experiments on the ADNI datasets showed that RELM with the feature selection approach can significantly improve classification accuracy of AD from MCI and HC subjects.

## 1. Introduction

Alzheimer's disease (AD) is a slow fatal neurodegenerative disease affecting people over the age of 65 years [1], while early-onset AD is also diagnosed before 65. The deposition of two abnormal protein fragments known as plagues and tangles in the brain causes the death of neuron cells. The hippocampus, where the memories are first formed, is the initially affected region by AD, and thus early symptoms of AD include memory problems resulting difficulties in word finding and thinking processes [2]. AD patients suffer from a lack of initiative, changes in personality or behavior in day-to-day functions at home, or at work, and in taking care of oneself, eventually, leading to death. The brain volume reduces dramatically through time and affects most of its functions with the progression of AD.

With the increase in the population of elderly people in developed countries, AD is going to be a major problem in socioeconomic implications. According to the recent report [3], it is expected that the number of affected people will be doubled in the next 20 years and one in two aged above 85 years will suffer from AD by 2050. Thus, accurate diagnosis of AD is very important, especially, at its early stage. Conventionally, the diagnosis of AD is performed by a neuropsychological examination in support of structural imaging. It is reported in [4] that (1) in the early stage of AD, degeneration of neurons takes place in the medial temporal lobe, (2) gradually affecting the entorhinal cortex, the hippocampus, and the limbic system, and (3) neocortical areas are affected at the final stage. Therefore, the study of medial temporal lobe atrophy (MTA), particularly in the hippocampus, the entorhinal cortex, and the amygdala provides the evidence of the progression of AD. Generally, MTA is measured in terms of voxel-based [5], vertex-based [6], and ROI-based [7] approaches. However, as the disease progresses, other regions in the brain are also affected. In such cases, whole-brain methods are preferred rather than a specific region-based method; then, the characterization of brain atrophy for differentiating AD and MCI patients can be performed more efficiently.

In recent years, major advances in neuroimaging have provided opportunities to study neurological-related diseases, resulting improvements in early and accurate detection of AD [5, 6, 8]. Magnetic resonance imaging (MRI) is more widely used in AD-related studies because of its noninvasive nature and lack of pain to patients. In addition, MRI provides an excellent spatial resolution and good contrast [5–7, 9]. Thus, several studies have used structural MRI- (sMRI-) based biomarkers to classify AD [10–19], which describes brain atrophy and change in the size of brain tissues. Similarly, functional MRI (fMRI) [20] can be utilized to characterize the hemodynamic response relevant to neural activity and functional/structural connectivity [21–23], which can be used to describe neurological disorders in the whole brain at the connectivity level. In this paper, we focused only on AD classification using sMRI. The intensity and stage of the neurodegeneration can be identified by the help of atrophy measured by sMRI [24]. Thus, sMRI-based feature extraction has attracted the attention for researchers of AD classification. These studies include morphometric methods such as region of interest (ROI)/volume of interest (VOI) grey matter voxels in the automatic segmentation of images [25] and the sMRI measurement of the hippocampus and the medial temporal lobe [26].

**Figure 1:**
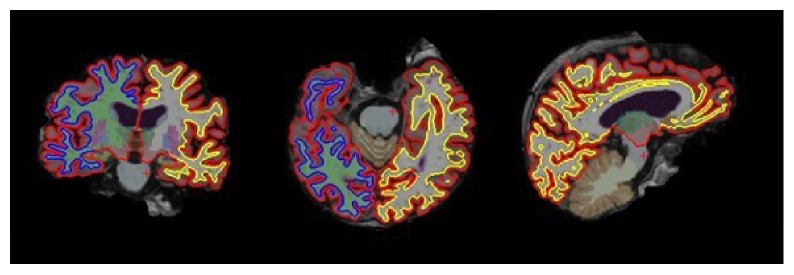
Segmentation of brain MR images for volumetric study.

Several machine learning techniques have been used to distinguish AD subjects from elderly control subjects using different biomarkers. The commonly used classifiers include support vector machine (SVM), artificial neural network (ANN), and other ensemble classifiers. Among them, SVM and the variants have been widely studied due to its relatively good accuracy and ability to deal with high-dimensional data. A SVM-type classifier (e.g., Magnin et al. [27]) begins with a learning stage from the training dataset consisting of well-characterized subjects with known states (i.e., labels for the subjects are given). Then, the classifier aims to maximize the margin of the training data by constructing the optimal separating hyperplane or a set of hyperplanes in a single- or higher-dimensional space. At a testing stage, classification is performed for test dataset based on the learned hyperplane(s). In general, three-dimensional (3D) T1-weighted MR images of each subject were automatically parcellated into ROIs. Grey matter from each ROI is extracted, as shown in Figure 1, as a feature for classification.

Zhang et al. [10] proposed a multimodal classification approach by utilizing multiple-kernel SVM based on the biomarkers including sMRI [18, 19], positron emission tomography (PET) [6], and cerebrospinal fluid (CSF) [28] to discriminate AD (or MCI) and normal control (NC) subjects. From the binary classification (i.e., AD versus NC and MCI versus NC) results, their proposed model could obtain a good accuracy for AD classification and an encouraging accuracy for MCI classification. Liu et al. [29, 30] proposed deep learning-based multiclass classification among normal controls (NC), MCI nonconverters (ncMCI), MCI converters (cMCI), and AD subjects based on 83 ROIs of sMRI images and the corresponding registered PET images. Stacked autoencoders (SAE) were used as unsupervised learning to obtain high-level features, and then softmax logistic regression was adopted as the classifier. While the experimental results showed reasonably good performance, it is still arguing that the denoising nature of SAE can increase the difficulty of suitable feature learning and thus it may be difficult for practical use. Li et al. [31] proposed fine-grained new features based on principle component analysis (PCA), stability selection, dropout, and multitask learning, where restricted Boltzmann machine (RBM) model was used as the deep learning architecture. 93 ROIs of MRI and PET images, together with CSF biomarkers, are used. Ye et al. [32] introduced conceptual machine learning-based multimodal data fusion approach using MRI, PET, genetic, CSF, demographic for AD-related research, and functional connectivity analysis. Recently, Rama et al. [33] proposed IVM-based classification approach for multiclass classification. In this method, only the subset of features from structural MRI was used as input to kernel logistic regression thus reducing the computational cost. This method used total 65 ROIs as features for training and testing and achieved the accuracy of up to 70% while classifying AD, MCI, and HC and 76.9% for binary classification of HC and AD [33].

While several approaches have been proposed for classification of different AD stages, with relatively small dataset, it is very difficult to extract effective information. This work focuses on comparing and presenting efficient classification approaches working robustly for a relatively small dataset. To this end, we present and compare three representative classifiers, with an efficient feature selection approach, including SVM, an import vector machine (IVM) and a regularized extreme learning machine (RELM) for the multiclass classification of different stages of AD progression.

## 2. Materials for Study

### 2.1. sMRI Dataset

Data used in preparation of this paper were obtained from the Alzheimer's disease neuroimaging initiative database (ADNI) (http://adni.loni.usc.edu/). The ADNI database was launched in 2003 as a public-private partnership. The primary goal of ADNI has been to test whether the serial MRI, PET, other biological markers, and clinical and neurophysical assessment can be combined to measure the progression of midcognitive impairment and the early AD.

### 2.2. Subjects

The ADNI dataset consists of more than 6000 subjects aged from 18 to 96. From it, we selected 214 subjects aged between 65 and 96. The selected participants met the criteria defined in the ADNI protocol. We constructed balanced dataset consisting of 214 subjects as follows:

1. 70 NC subjects: 33 males, 37 females; age ± SD= 76.3 ± 5.4 years, range = 60–90 years; mini-mental state estimation (MMSE) score = 29.2 ± 1.0, range = 25–30.
2. 74 MCI subjects who had not converted to AD within 18 months: 38 males, 36 females; age ± SD = 74.5 ± 7.2 years, range = 58–88 years; MMSE score = 27.2 ± 1.7, range = 24–30.
3. 70 AD subjects: 39 males, 31 females; age ± SD = 76.0 ± 7.3 years, range = 55–91 years; MMSE = 23.2 ± 2.0, range = 18–27.

Table 1 shows a summary of demographic status of the selected subjects.

All structural MR (sMR) scans used in this work were acquired from 3T scanners. The main focus of this work was to elaborate the supervised multiclass classification among NC, MCI, and AD based on different classifiers. Thus, to obtain unbiased estimates of the classifier performance, the selected subjects were randomly split up into two groups of the training dataset and the testing dataset. The algorithms were trained on a training set, and the performances of the diagnostic sensitivity and specificity together with accuracy were evaluated on an independent test dataset. The division process considers balanced age and sex distributions.

### 2.3. Preprocessing of sMRI Data

**Figure 2:**
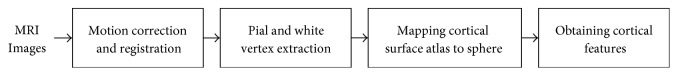
Preprocessing steps of sMRI images.

We used a fully automated pipeline of the FreeSurfer 5.3.0 software package for reconstruction and volumetric segmentation from all the sMRI images and extracted the pattern of useful data. The software performs a series of preprocessing operations with the FreeSurfer's recon-all processing pipeline on the original sMRI data as shown in Figure 2. The preprocessing steps include motion correction, T1-weighted image averaging, registration of volume to the Talairach space, skull striping with a deformable template model. The white surface and the pial surface are generated for each hemisphere using encoding the shape of the corpus callosum and pons in the Talairach space and following the intensity gradients from the white matter. The accurate matching of the morphologically homologous cortical locations across subjects was estimated using the mapping of the atlas based on a cortical surface to a sphere aligning the cortical patterns. Cortical thickness at each vertex of the cortex is denoted by the average shortest distance between white and pial surfaces. The area of every triangle in a standardized spherical surface tessellation provides the surface area. Similarly, the registration surface based on the folding pattern was used to compute the local curvature. The method developed by Schaer [34] was used to measure the folding index over the whole cortical surface. All the extracted features are explained in terms of feature measures as in Table 2.

### 2.4. Details of the sMRI Data

**Figure 3:**
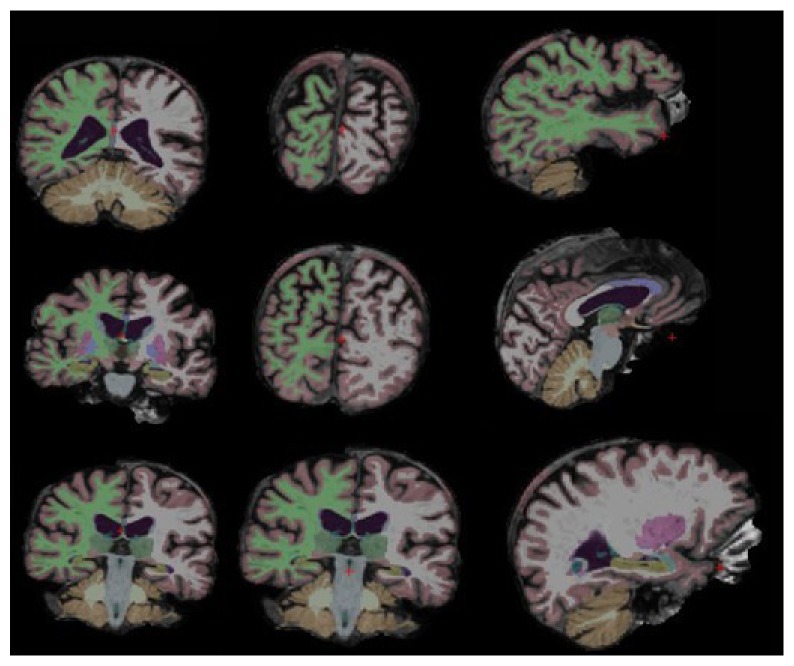
Block brain regions selected for AD classification using sMRI images.

We perform binary classification for NC versus AD and multiclass classification using the one-versus-all (OVA) class setting for NC, MCI, and AD. For the subjects and groups chosen as in Table 2, volumetric features, fM5, in Table 2, were used for the study, and for each feature, we computed the grey matter tissue volume from the individual subject's sMRI image. Block brain regions selected for the classification are shown in Figure 3. Each tissue is discriminated from other tissues by using color code defined by FreeSurfer software package. The left column presents the coronal view followed by the saggital view in the middle column and the axial view at the rightmost column. We followed neurological convention for the view. All sMR scans used in this paper were acquired from 3T scanners.

## 3. Proposed Methods: Classification of Stages of AD Progression

**Figure 4:**
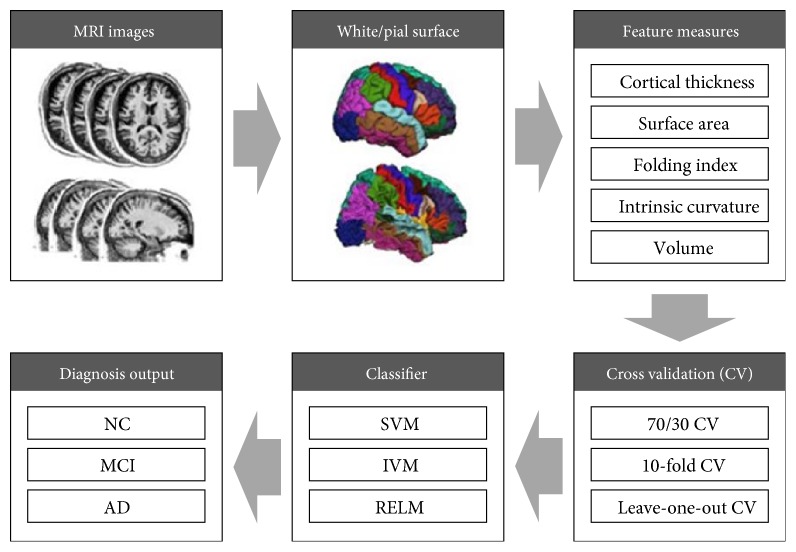
Block diagram of automatic diagnosis system.

We used the three representative machine-learning classification algorithms, SVM, IVM, and RELM. The stepwise block diagram of the classification of stages of AD progression is shown in Figure 4.

### 3.1. Efficient Feature Selection

In neuroimaging analysis, the number of features per subject can be very high compared to the number of subjects, which is commonly referred to as the curse of dimensionality. We perform an efficient feature selection method based on PCA which is a method widely used to reduce the dimensionality of a high-dimensional (imaging) data [25]. As the result, the most information representative dimensions are kept while the least important ones are excluded. PCA generates new features which are a linear combination of the initial features and maps each instance of the given dataset present in a *d*-dimensional space to a *k*-dimensional subspace such that *k* < *d*. The set of *k* new dimensions generated are called the principal components (PCs), and each PC is directed towards maximum variance excluding the variance already accounted for in all its preceding components. Subsequently, the first component covers the maximum variance, and each component that follows it covers a lesser value of variance. PCs can be represented as

(1) $$\begin{array}{c} {PC}_{i}=a_{1}X_{1}+a_{2}X_{2}+\cdots +a_{d}X_{d}, \end{array}$$

where PC_*i*_ is the *i*th PC, *X*_*j*_ is the original feature *j*, and *a*_*j*_ represents the numerical coefficient for *X*_*j*_.

### 3.2. SVM Classifier

SVM [35] is basically a binary classifier which is useful for the classification of both separable and nonseparable data. It has been used in the neuroimaging field and considered as one of the most popular machine learning tools in the neuroscience domain in the last decade. It is a supervised classification algorithm and finds the optimal hyperplane that separates both classes with maximum margin from support vectors during the training phase. For the testing of new data points, the classifier's decision is based on the estimated hyperplane. For the linearly separable patterns, linear SVM is used. However, linear SVM cannot guarantee better performance in complex cases with nonseparable patterns. In such scenario, linear SVM is extended using kernel trick. The input patterns are mapped into a higher dimensional space using linear and nonlinear functions known as kernels. Linear and nonlinear radial basis function (RBF) kernels are widely used SVM kernels.

### 3.3. IVM Classifier

The fundamental principle of IVM proposed by Zhu and Hastie [36] is built on kernel logistic regression (KLR). It has not merely performed well in the binary classification as SVM, and it can be naturally generalized to the multiclass classification. Thus, we begin with the explanation of logistic regression. Let *x*_*i*_ = (*x*_1_,…,*x*_*n*_)^*T*^ represent observed samples with class labels *y*_*j*_ ∈ *C*  {*j* = 1,…, *K*} pattern classes. The training set is represented as(*x*_*i*_, *y*_*j*_), *i* = 1,…, *n*. For the binary class problem, where input samples *x*_*i*_ are independent and identically distributed, the conditional class posterior probability *P*_*i*_(*y*_*i*_/*x*_*i*_; *w*) is estimated using the following logistic regression model:

(2) $$\begin{array}{c} P_{i}\left(\frac{y_{i}}{x_{i};w}\right)=\frac{1}{1+\exp\left(w^{T}x_{i}\right)}. \end{array}$$

The logistic regression predicts the class based on probabilities which are either *p* for *y*_*i*_ = 1 or 1 − *p* for *y*_*i*_ = 0. Thus, we can express the cost function of logistic regression as

(3) $$\begin{array}{c} Q_{0}\left(w\right)=\overset{n}{\underset{i=1}{\prod }}p{\left(x_{i}\right)}^{y_{i}}{\left(1-p\left(x_{i}\right)\right)}^{1-y_{i}}. \end{array}$$

In order to fit the parameters for the given model by training the given data points, we try to find the parameter *u* that minimizes *Q*_0_. As a result, *u* is selected, which is most likely to generate the labels as the same as in the training set. The minimization can be obtained by using the gradient and the Hessian. In order to prevent overfitting, one may introduce a prior over the parameters and optimize

(4) $$\begin{array}{c} Q\left(w\right)=Q_{0}\left(w\right)+\frac{\lambda }{2}w^{T}Lw. \end{array}$$

Therefore, the iteration scheme could simply be formulated with the Newton-Raphson iteration method. To extend the linear model to a nonlinear one, the original features *x*_*n*_ are transformed into the higher dimensional space *k*_*n*_ using a kernel function

(5) $$\begin{array}{c} k_{nn}=k\left(x_{n},x_{n}\right). \end{array}$$

The model of kernel logistic regression now presumes the a posteriori probabilities are given by

(6) $$\begin{array}{c} P_{nc}\left(w\right)=\frac{\exp\left(w_{c}^{T}k_{n}\right)}{\underset{c}{\sum }\exp\left(w_{c}^{T}k_{n}\right)}, \end{array}$$

with *k*_*n*_ as the *n*th column of the kernel matrix *K*, and the unknown parameter *w* = […, *w*_*c*_,…] refers to *c* classes. The parameters are determined in an iterative way by optimizing the regularized objective function. One of the limitations of the standard KLR is that all possible training samples are used to evaluate the kernel function, thus increasing the computational complexity and the memory requirement for large datasets. Meanwhile, the complexity of the classifier can be controlled by enforcing the sparseness in the learning model. The sparse kernel machine uses only the kernel function evaluated at a subset of the training data points for prediction of new inputs. The most common methods to implement sparseness are by introducing a suitable prior or by a subset selection. One of the popular examples for sparse kernel machine is SVM, which only supports that vectors are used to predict new inputs. The main idea of incorporating sparseness into KLR is to select a subset *v* of *V* feature vectors out of the training set *T*. Thus, the kernel matrix only consists of the selected a subset *v* of important kernels *k*_*v*_ from all samples *T*. IVM uses a smaller fraction of training data to realize the sparse KLR. The subset is determined by a greedy manner. This method begins with empty set *v* and then constructs the set of import vectors by successively adding data samples. The construction process of sets stops once the convergence criterion is reached. The convergence criterion is used by the ratio *ε* = |*Q*_*t*_ − *Q*_*t*−Δ*t*_|/|*Q*_*t*_| with a small integer Δ*t* such as the regularization, and the kernel parameter *ε* defines the threshold for excluding import vectors. Consequently, this criterion influences the sparseness of the model.

### 3.4. RELM Classifier

Single hidden-layer feed forward neural networks (SLFNs), such as the back propagation (BP) learning algorithm, are widely used machine learning techniques for research in various fields. These methods minimize the cost function to maintain the accuracy within an acceptable range by searching the specific input weights and hidden layer biases, which leads to increase in computational cost. Extreme learning machine (ELM) is a learning algorithm implemented without iteratively tuning the artificial hidden nodes, thus decreasing the computation time [37]. ELM is an effective solution for SLFNs. The SLFN with *L* hidden nodes and an activation function *g(x)* is expressed as

(7) $$\begin{array}{c} Y_{L}\left(x\right)=\overset{L}{\underset{i=1}{\sum }}\beta_{i}h_{i}\left(x\right)=h\left(x\right)\beta_{i}, \end{array}$$

where *β* = [*β*_1_,…,*β*_2_]^*T*^ is an output weight matrix between the hidden nodes and output nodes. *h*_*i*_(*x*) is the hidden node output. Unlike SVM and other BP-based methods, the parameters of the hidden layer such as the input weight *w_i_* and the hidden layer biases *b_i_* need not to be tuned and can be generated randomly before the training samples are acquired. Given *N* training samples {(*x*_*j*_, *t*_*j*_)}_*j*=1_^*N*^, ELM solves the learning problem by minimizing the error between *t*_*j*_ and *Y*_*j*_:

(8) $$\begin{array}{c} \left\|H\left(w_{1},\ldots ,w_{\overset{˜}{N}},b_{1},\ldots ,b_{\overset{˜}{N}}\right)\hat{\beta }-T\right\|=\underset{\beta }{\min}\;\left\|H\hat{\beta }-T\right\|, \end{array}$$

where

(9) $$\begin{array}{c} \begin{array}{c} H\left(w_{1},\ldots ,w_{\overset{˜}{N}},b_{1},\ldots ,b_{\overset{˜}{N}}\right)=\left[\begin{array}{ccc} g\left(w_{1}\cdot x_{1}+b_{1}\right) & \cdots & g\left(w_{L}\cdot x_{1}+b_{L}\right)\\ \vdots & \cdots & \vdots \\ g\left(w_{1}\cdot x_{N}+b_{1}\right) & \cdots & g\left(w_{L}\cdot x_{N}+b_{L}\right) \end{array}\right],\\ \beta =\left[\begin{array}{c} \beta_{1}^{T}\\ \vdots \\ \beta_{L}^{T} \end{array}\right],\\ T=\left[\begin{array}{c} t_{1}^{T}\\ \vdots \\ t_{L}^{T} \end{array}\right]. \end{array} \end{array}$$

Here, *H* is called the hidden layer output matrix. The output weights *β* can be calculated as

(10) $$\begin{array}{c} \beta =H^{+}T, \end{array}$$

where *H*^+^ is the Moore-Penrose generalized inverse of the matrix *H* with the advantage of speed. ELM is well-suited for the tasks related to neuroimaging and big data for the classification of binary and multiclass settings. However, the decrease in computation time increases the error in the output, thus decreasing the accuracy. To increase the accuracy, ELM is combined with sparse representation. This hybrid algorithm performs classification in two fundamental steps [38–40]. In the first stage, the ELM network is trained with the conventional training approach. However, in the testing stage, reliability-based classification is used. In reliability-based classification, the ELM classifier is employed if the test data is correctly classified; otherwise, the sparse representation-based classification is used [41]. Additionally, a regularization term is added to improve generalization performance and make the solution more robust. Finally, the output weight of the RELM can be expressed as

(11) $$\begin{array}{c} \beta ={\left(\frac{I}{C}+H^{T}H\right)}^{-1}H^{T}T. \end{array}$$

## 4. Experimental Results and Analysis

### 4.1. Permutation Testing

Permutation testing can be applied to assess the statistical significance of the classifier [42]. The assessment proceeds with the selection of the test statistic of the classifier and assigns random labels to the classifier by permuting the class labels for the training dataset. Permutation testing involves performing cross validation (CV) on data for which the diagnostic label has been randomly permuted. This leads to a distribution of classification results under the null hypothesis that the classifier cannot accurately predict the clinical labels from the data. The *p* value of the permuted prediction rate against the prediction rate with the original data labels indicates the significance of the classifier. In this work, we used 70/30 CV, 10-fold CV, and leave-one-out (LOO) CV methods. Experiments for both binary and multiclass classification were carried out with the same setup.

### 4.2. Performance Evaluation Methods

We evaluated the performance of the proposed algorithm with the IVM, SVM, and RELM classifiers for each specific test including binary and multiclass classification tasks. The performance of the binary classification for the two subjects S1 and S2 can be visualized in a form of a confusion matrix as shown in Table 3. Diagonal elements of the matrix indicate the number of correct predictions by the classifier. The elements can be further divided into true positive (TP) and true negative (TN), which represent correctly identified controls. Similarly, the number of wrongly classified subjects may be represented by false positive (FP) and false negative (FN).

The accuracy measures the proportion of examples that are correctly labeled by the classifier.

(12) $$\begin{array}{c} ACC=\frac{TP+TN}{TP+TN+FP+FN}. \end{array}$$

However, for dataset with very unbalanced class distribution, accuracy in (12) may be a misleading performance metric. Thus, two performance metrics known as sensitivity and specificity are also used.

(13) $$\begin{array}{c} SEN=\frac{TP}{TP+FN} \end{array}$$

(14) $$\begin{array}{c} SPE=\frac{TN}{TN+FP}. \end{array}$$

The sensitivity in (13) measures the rate of true positives while the specificity in (14) measures the rate of true negatives. The performance metrics for the multiclass classification are easily extended as the averaged ones on the OVA setting.

### 4.3. Binary Classification: Results and Analysis

The experimental results of binary classification (NC versus AD) are shown in Table 4, and those with feature selection are in Table 5. 141 subjects were randomly selected for the binary classification. Initially, we randomly segregated the training and testing dataset and used the first 111 randomly chosen subjects from each group for training and the remaining 30 subjects for testing the classifier. Similarly, in 10-fold cross-validation, all 141 subjects were randomly divided into equally sized subsets, that is, 10% testing subjects and 90% training subjects, for each of the 10-fold sets of the CV. In addition, for the nested validation, we repeated the classification experiment 10 times in the case of the 10-fold CV and leave-one-out CV, and 100 times in the case of the conventional 70/30 CV, to ensure the robustness of the classification results. The mean accuracy of all the repetitions was calculated by the final results.

**Figure 5:**
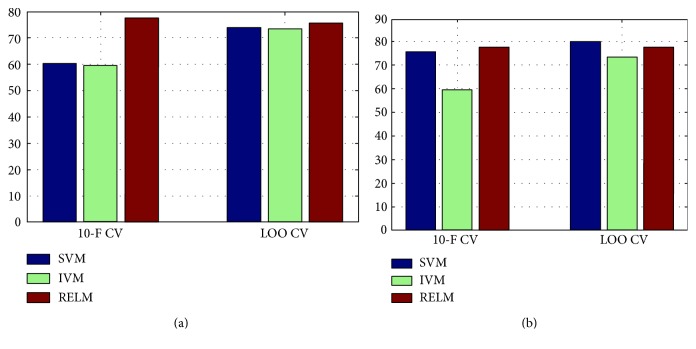
Performance comparison of binary classification in terms of accuracy: (a) binary classification and (b) binary classification with feature selection.

In Table 4 showing the baseline performance of different classifiers, all classifiers except IVM obtained good performance. There was no substantial difference, in terms of accuracy, between the results obtained with IVM and SVM, and RELM is better than the others in 10-fold CV; however, SVM is better than RELM in LOO CV. For the feature selection, the datasets of size *n* × *d* were mapped to the given *k* principal component framework and transformed into the dataset of size *n* × *k*, where *n* is the number of subjects and *d* is the original number of features. The dataset originally consists of total 54 features. The number of PCs represented as *k* ranging from 2 to 20, with an incremental offset of two, was checked and the best one was selected for each classifier. From repeated simulations, we achieved the generally good accuracy when the value of *k* is set to 10. As shown in Table 5, by adopting feature selection, the similar performance characteristic was observed in terms of accuracy. From Figure 5, it is easily observed the effectiveness of the feature selection approach in 10-fold CV and LOO CV cases. It is meaningful that from our repeated simulations, we found that the results of 70/30 CV case, which is a widely used setting, are not stable (i.e., it has large variance with different trials) mainly due to overfitting problems, and thus, the results were not listed in this work.

### 4.4. Multiclass Classification: Results and Analysis

**Figure 6:**
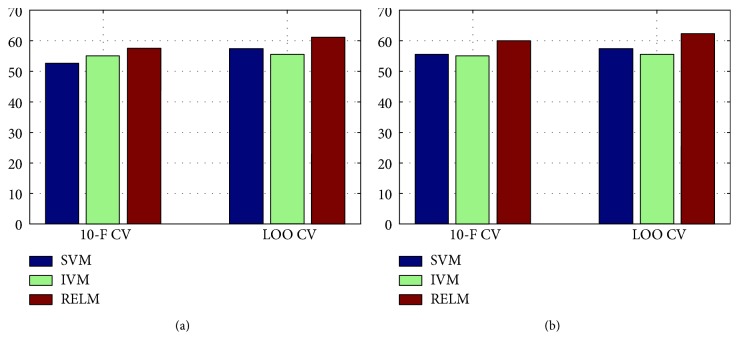
Performance comparison of multiclass classification in terms of accuracy: (a) multiclass classification and (b) multiclass classification with feature selection.

For multiclass classification, we adopted all labeled 214 subjects in Table 1. The same subjects were used in binary classification, and we adopted three CV methods. From Tables 6 and 7, it is easily observed that RELM outperforms SVM and IVM in terms of accuracy. From Figure 6, we also could see the effectiveness of the feature selection approach in 10-fold CV and LOO CV cases. Similar to binary classification cases, on the 70/30 CV case, we obtained the experimental results with large variance, and thus, they were also excluded from the analysis. From the results, it is naturally driven that multiclass classification (which is the general form in clinical diagnosis of AD) assisted by RELM is effective compared to the other considered representative classifies.

### 4.5. Discussion on the Results

It has been known that in many problem tasks, IVM generally performs similar with SVM in terms of accuracy and provides probabilistic output. From our experiments, we could confirm that SVM generates better accuracy compared to IVM, which is mainly attributed to the robustness of SVM to outliers. The main impetus of this study was to compare representative classifiers, SVM, IVM, and RELM for binary and multiclass classification tasks. Trivially, the accuracy of the binary classification cases was higher than the corresponding multiclass classification cases. Also, the experimental results on large dataset of 214 subjects verified that RELM-based AD diagnosis framework (significantly) outperform the others with higher accuracy. To the best of our knowledge, this is the first study in which the RELM framework was used for multiclass classification on sMRI data obtained from the ADNI dataset. To classify the effectiveness of feature selection in combination with the classifiers, we utilized the PCA-based feature selection method as an efficient approach to validate its efficiency. It selects features that represent higher degrees of significance based on the internal linear SVM-based classification scores, and thus, it has the possibility of making classification significantly more accurate. The experimental results also support that such adoption of feature selection can be beneficial to improve the accuracy of the classifiers, SVM, IVM, and RELM. From the noteworthy results, we could conclude that the approaches for the stage classification can be used as an effective assistive tool for the establishment of a clinical diagnosis.

## 5. Conclusions and Future Work

The early diagnosis of AD and MCI is essential for patient care and research, and it is widely accepted that preventive measures plays an important role to delay or alleviate the progression of AD. For the classification task of different stages of AD progression, the smaller number of training samples and the larger number of feature representations are the major challenges. In this study, we investigated SVM, IVM, and RELM for the classification problem. In IVM, only the subsets of the input vectors of KLR are selected by minimizing the regularized cost function to reduce computation time. RELM is an effective solution for SLFNs implemented without iteratively tuning the artificial hidden nodes and adopts reliability-based classification where ELM is adopted if the test data is correctly classified, and sparse representation is selected for the other cases. Experiments on the ADNI dataset showed that RELM-based classifier could significantly improve accuracy in both binary and multiclass classification tasks. In addition, we could observe that adoption of the PCA-based feature selection could improve the accuracy slightly. While this study is focusing on the stage diagnosis of AD progression using sMRI alone, further study is still being carried out to improve the accuracy by elaborating the classifiers, possibly using a model ensemble approach, and feature selection. Also, the studies of adding more modalities such as fMRI and PET in combination with sMRI are also one of our future researches.

## Figures and Tables

**Table 1:** Summary of subject's demographic status.

	NC	MCI	AD
Number of subjects	70	74	70
Average age	76.3	74.5	76.0
Average education points	16.19	15.96	15.53
MMSE	29.2 ± 1.0	27.2 ± 1.7	23.2 ± 2.0

**Table 2:** Feature measures and cortical feature index information.

Feature measure (fM)	Feature measure type	Indices of cortical feature
fM1	Mean cortical thickness	1–64
fM2	Surface area	65–128
fM3	Folding indices	193–256
fM4	Mean curvature indices	193–256
fM5	Volume	257–320

**Table 3:** Confusion matrix.

True class	Predicted class	
	S1	S2
S1	TP	FN
S2	FP	TN

**Table 4:** Performance of binary classification.

CV method	Classifier	Performance metrics		
		ACC (%)	SEN (%)	SPEC (%)
10-fold CV	SVM	60.10	**74.63**	**88.81**
	IVM	59.50	62.30	62.85
	RELM	**77.30 (** **p** < 0.0001**)**	62.12	79.85
LOO CV	SVM	**78.01**	**75.81**	**79.12**
	IVM	73.36	70.97	75.95
	RELM	75.66 (*p* < 0.0001)	72.13	77.22

**Table 5:** Performance of binary classification with feature selection.

CV method	Classifier	Performance metrics		
		ACC (%)	SEN (%)	SPEC (%)
10-fold CV	SVM	75.33	**77.51**	61.20
	IVM	60.20	62.50	81.10
	RELM	**76.61 (** **p** < 0.0001**)**	61.70	**90.63**
LOO CV	SVM	**80.32**	83.37	78.82
	IVM	74.47	**87.10**	64.56
	RELM	77.88 (*p* < 0.0001)	68.85	**83.54**

**Table 6:** Performance of multiclass classification.

CV method	Classifier	Performance metrics		
		ACC (%)	SEN (%)	SPEC (%)
10-fold CV	SVM	52.63	42.74	56.77
	IVM	54.90	46.18	**60.82**
	RELM	**57.56**	**56.34**	56.73
LOO CV	SVM	57.40	55.25	58.62
	IVM	55.50	**60.78**	52.22
	RELM	**61.20**	50.00	**66.89**

**Table 7:** Performance of multiclass classification with feature selection.

CV method	Classifier	Performance metrics		
		ACC (%)	SEN (%)	SPEC (%)
10-fold CV	SVM	56.60	50.59	56.38
	IVM	56.14	40.16	64.83
	RELM	**59.81**	58.25	58.82
LOO CV	SVM	58.30	**57.12**	60.32
	IVM	56.80	64.71	49.56
	RELM	**61.58**	54.00	**62.25**
